# Plant Glycine-Rich Proteins in Stress Response: An Emerging, Still Prospective Story

**DOI:** 10.3389/fpls.2018.00302

**Published:** 2018-03-08

**Authors:** Magdalena Czolpinska, Michal Rurek

**Affiliations:** Department of Molecular and Cellular Biology, Institute of Molecular Biology and Biotechnology, Adam Mickiewicz University, Poznań, Poland

**Keywords:** GRPs, conserved segments, temperature stress, wounding, osmotic stress, drought, oxidative stress, biotic stress

## Abstract

Seed plants are sessile organisms that have developed a plethora of strategies for sensing, avoiding, and responding to stress. Several proteins, including the glycine-rich protein (GRP) superfamily, are involved in cellular stress responses and signaling. GRPs are characterized by high glycine content and the presence of conserved segments including glycine-containing structural motifs composed of repetitive amino acid residues. The general structure of this superfamily facilitates division of GRPs into five main subclasses. Although the participation of GRPs in plant stress response has been indicated in numerous model and non-model plant species, relatively little is known about the key physiological processes and molecular mechanisms in which those proteins are engaged. Class I, II, and IV members are known to be involved in hormone signaling, stress acclimation, and floral development, and are crucial for regulation of plant cells growth. GRPs of class IV [RNA-binding proteins (RBPs)] are involved in alternative splicing or regulation of transcription and stomatal movement, seed, pollen, and stamen development; their accumulation is regulated by the circadian clock. Owing to the fact that the overexpression of GRPs can confer tolerance to stress (e.g., some are involved in cold acclimation and may improve growth at low temperatures), these proteins could play a promising role in agriculture through plant genetic engineering. Consequently, isolation, cloning, characterization, and functional validation of novel GRPs expressed in response to the diverse stress conditions are expected to be growing areas of research in the coming years. According to our knowledge, this is the first comprehensive review on participation of plant GRPs in the response to diverse stress stimuli.

## Introduction

Plants have evolved a number of adaptive stress responses that operate at morphological, physiological, biochemical, and molecular levels. These strategies depend on a plethora of diverse signaling pathways, involving a broad spectrum of molecules, including enzymes, transcription factors, and other proteins associated with plant defense systems. Such strategies seem to be crucial because plants are sessile organisms constantly exposed to environmental stimuli (Al-Whaibi, 2011; Smékalová et al., 2014; Pan et al., 2016). Under the influence of multiple stressors, distinct adaptive mechanisms are more active than is observed in the case of a single stressor (Miao et al., 2015; Pandey et al., 2015).

Abiotic stress, such as decreased or elevated temperature, can seriously limit plant growth and productivity and, in consequence, global food production (Miao et al., 2015; Guo et al., 2016; Sah et al., 2016; Wang et al., 2016; Rihan et al., 2017). It is increasingly important to reduce losses in food production. To understand the complex mechanisms affecting plant growth and development under stress-inducing conditions, a number of studies on economically and ecologically important species are currently underway. The characterization of novel stress-responsive proteins, including glycine-rich proteins (GRPs), will provide substantial insight into stress tolerance mechanisms and will facilitate plant genetic improvement through the production of genotypes with enhanced stress tolerance or rapid growth and development under sub-optimal environmental conditions (Miao et al., 2015; Pandey et al., 2015; Wang et al., 2016; Rihan et al., 2017).

In this review, various classes of GRPs are described, with special focus on class IV GRPs, which are RNA-binding proteins (RBPs) known to be involved in plant defense mechanisms. To our knowledge, this is the first complex compilation on the participation of plant GRPs in various stress responses. Such proteins are involved in the regulation of diverse steps in RNA post-transcriptional processing, including splicing and polyadenylation, which are believed to play a crucial role in responses to a variety of detrimental conditions (Yang et al., 2014). GRPs also belong to the most important proteins involved in plant defense pathways; despite this, little is known about their function or the relationship between their structure and function. In the current review, we will focus on the structure and crucial functions of GRPs. Despite some gaps in current knowledge, this study presents novel and interesting evidence for specific involvement of GRPs in stress responses and highlights the broad spectrum of activities performed by GRPs and their modes of action.

## Characteristics of the GRP superfamily

**Figure 1:**
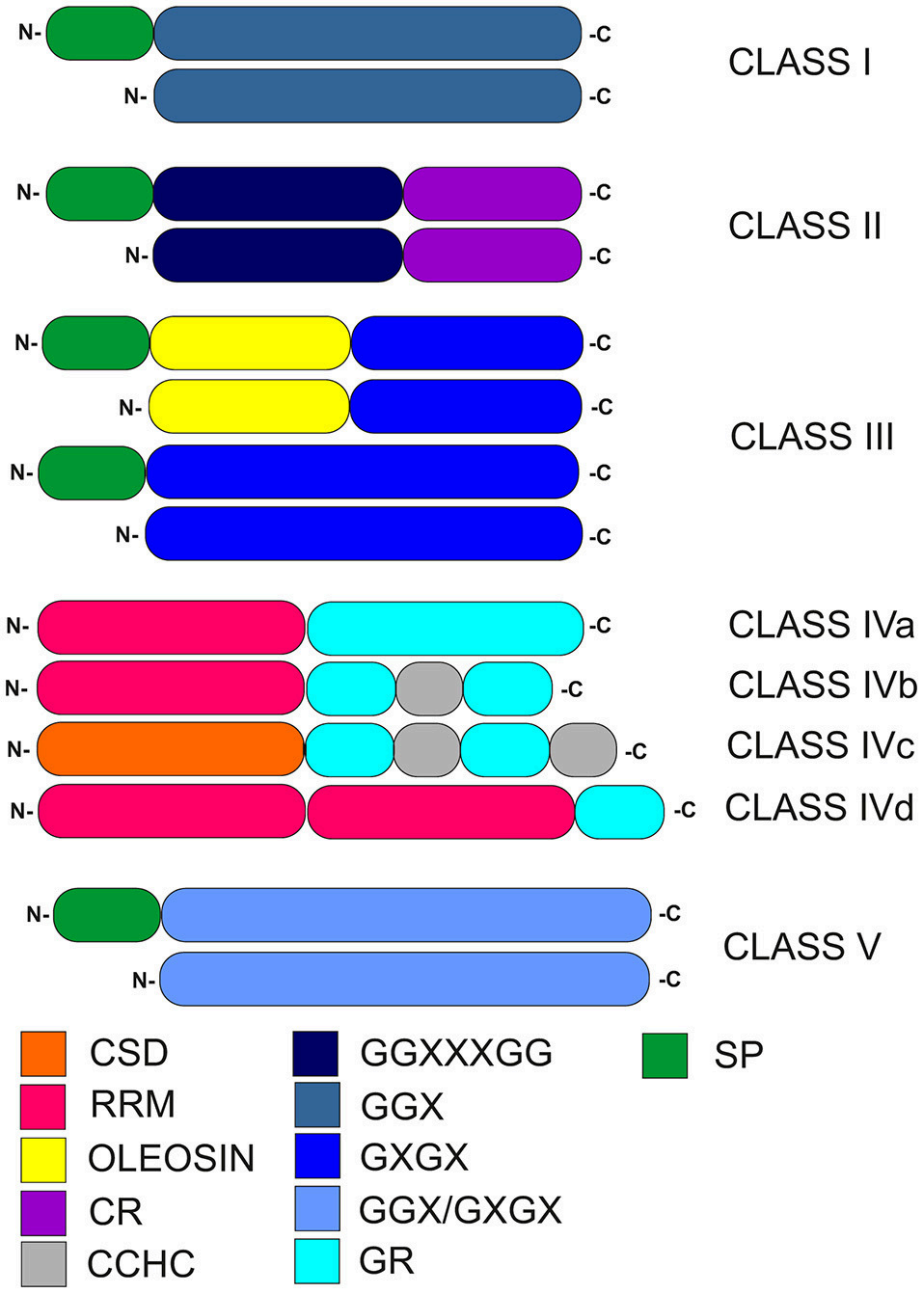
Classification of plant glycine-rich proteins. CSD, cold-shock domain; RRM, RNA-recognition motif; oleosin, oleosin-conserved domain; CR, cysteine-rich domain; CCHC, zinc-finger; GR, glycine-rich domain; GGX, GGXXXGG, GXGX, GGX/GXGX, glycine-rich repeats (G-Gly, X-any amino acid); SP, signal peptide.

GRPs are known to be involved in plant defense systems induced by abiotic and biotic stress, and are characterized by a high glycine content (up to 70%) and the presence of glycine-containing motifs composed of repetitive amino acid residues (Ortega-Amaro et al., 2015). The general structure of GRPs makes it possible to divide them into five classes (Figure 1, Table 1). This categorization is based on the arrangement of glycine repeats and presence of conserved motifs within particular GRPs (Mangeon et al., 2010).

**Table 1:** Classification of glycine-rich proteins basing on structural features.

**GRP classes**	**Characteristic features**
Class I	Signal peptide followed by high glycine-content region with (GGX)*_*n*_* repeats
Class II	Signal peptide and presence of a characteristic cysteine-rich C-terminal domain
Class III	Signal peptide and contain lower glycine content (in comparison to other GRPs classes), the oleosin domain is the signature motif for their sub-group
Class IV	RNA-binding GRPs, glycine-rich domain with RNA-recognition motif (RRM) or a cold shock domain (CSD), CCHC zinc-fingers might be also present in their structure, four sub-groups: (**IVa**) RRM motif besides the glycine-rich domain, (**IVb**) single RRM and CCHC zinc-finger motif, (**IVc**) cold shock domain and two or even more zinc-fingers, (**IVd**) two RRM motifs
Class V	Signal peptide followed by GGX/GXGX motif or only GGX/GXGX motif without signal peptide

Selected members of GRP classes I–III contain N-terminal signal peptide. Notably, such signal peptide in class I GRPs is followed by a region extremely rich in glycine and containing (GGX)_n_ repeats. In class II, two important regions are present, the first one containing [GG(X)_3_GG]_n_ glycine-rich repeats and the second one composed of a specific cysteine-rich motif at the C-terminus. The structurally heterogeneous class III is characterized by a substantially lower glycine content, compared to other GRP classes. It contains (GXGX)_*n*_ repeats, which are accompanied (in selected members) by an adjacent N-terminal oleosin-conserved domain. Class IV, which are also described as RNA-binding GRPs, contains numerous glycine-rich domains. A characteristic feature of class IV of GRPs is presence of a glycine-rich region at the C-termini and up to three N-terminal RNA recognition motifs (RRMs; Xu et al., 2014). This class is further subdivided into four subclasses (denoted IVa, IVb, IVc, and IVd). Members of subclasses IVa, IVb, and IVd contain RRMs, while the cold-shock domain (CSD) is present only in IVc subclass. In subclasses IVa, IVb, and IVd, one of RRMs (like the CSD motif within IVc subclass) is N-terminal. Subclasses IVb and IVc contain CCHC zinc-finger motifs in their secondary structure. Class V is characterized by mixed patterns of (GGX)_*n*_/(GXGX)_*n*_ repeats and a relatively high glycine content (Mangeon et al., 2010). Recently, distinct GRP superfamily members named RZs proteins, were described as a subgroup harboring characteristic internal CCHC-type-zinc finger motifs (Xu et al., 2014).

The role of glycine-rich domains in class IV GRPs has not been precisely established; on the physiological level, it is presumably involved in the cold tolerance and acclimation development. The length of glycine-rich domain is positively associated with stress tolerance. This domain containing zinc finger play a key role in nucleic acid-binding activity as well as RNA chaperoning (Nomata et al., 2004; Kim J. S et al., 2007). Hanano et al. (1996) performed deletion analysis of the C-terminal glycine-rich domain which showed that it is essential for RNA binding. Glycine-rich domain is more important than other functional domains of GRPs, which control protein conformation that are key for GRPs activity. It was predicted that the glycine-rich domain might be involved in interactions with numerous partners (Hanano et al., 1996). Further data on the diverse cellular functions of GRPs will be presented in section Cellular Activity of GRPs.

Recently, it has been suggested that GRPs in fact do not represent a distinct protein superfamily, but a group of proteins sharing only some repetitive structural motifs. This suggestion is based on their broad structural diversity, wide variety of modes of action, and their differing subcellular localization (Kar et al., 2012). According to The UniProt Consortium (2017), and SUBA databases predictions (Tanz et al., 2013) members of classes I and II were either predicted to be present in the cytosol and nucleus, or secreted and localized in the extracellular space. Class III was predicted to be secreted or present in the vicinity of the cell membrane. Class IVa is expected to be present in the cytosol, cell wall, Golgi, organelles, while classes IVb and IVc—seems to be restricted mainly to the nucleus. However, some *Arabidopsis* GRPs, particularly class IVa members (Supplementary Table 1a) could putatively be targeted to the mitochondria.

## Expression pattern of *GRP* genes and functional aspects

### Plant *GRP* genes and their co-expression pattern

In Supplementary Table 1a, general characteristics of the most important *Arabidopsis* GRPs and their genes is shown, based *inter alia* on TAIR data (Berardini et al., 2015). Genes encoding members of this superfamily are present in a number of seed plant genomes [e.g., eucalyptus (*Eucalyptus* sp.), rice (*O. sativa*), and *Arabidopsis*]. Data from PhytoMine service (Goodstein et al., 2012) and analyses of plant whole genomes and transcriptomes facilitated the identification of more than 150 distinct genes coding for GRPs in sugarcane (*Saccharum officinarum*), *Eucalyptus, Arabidopsis*, and rice, among other plant species (Mangeon et al., 2009, 2010; Supplementary Table 3c).

According to the data from ThaleMining repository (for the ARAPORT project) as well as ATTED-II database (Aoki et al., 2016), a plethora of genes co-expressed with *Arabidopsis GRP* genes as well as potential interacting partners were revealed. Co-expressed genes comprise genes coding for other GRPs as well as for other stress-related proteins, including LEA proteins and cellular chaperones and chaperonins, cell-wall associated kinases, leucine-rich receptor-like protein kinases, and other proteins (Supplementary Tables 3a,b).

### Cellular activity of GRPs

Owing to interaction patterns (chapter Plant GRP Genes and Their Co-Expression Pattern), GRPs are expected to participate in post-transcriptional regulation of gene expression; however, they display higher *in vitro* affinity to particular ribohomopolymers (Kim et al., 2005). In general, GRPs exhibit chaperonin activity toward nucleic acids, primarily RNA (Kwak et al., 2011).

Emerging evidence suggests that class I and II members are crucial for the positive regulation of plant cell and organ growth; they are also active components of the plant cell wall. Park et al. (2001) reported that class II of GRPs interact with cell wall-associated kinases, thereby initiating the recognition of environmental stimuli and participating in signal transduction. It has been demonstrated that GRPs are responsible for blocking tobamovirus infection (Mangeon et al., 2010). Moreover, GRPs are known for their anti-fungal and anti-bacterial activity, and this activity may allow them to protect plants from the cellular damage resulting from the action of the biotic stress. For instance, pumpkin (*Cucurbita pepo*) cucurmoschin peptide displayed antifungal activity against *Botrytis cinerea, Fusarium oxysporum*, and *Mycosphaerella oxysporum*. Another example of the specific biological activity of GRPs has been provided, namely their activity against Gram-negative bacteria (Cândido Ede et al., 2011). Evidence for the participation of those proteins in plant defense came from the analysis of molecular signals exchanged during interactions between *Arabidopsis* and *Pseudomonas syringae* (Fu et al., 2007).

In developmental processes such as pollen hydration and its recognition, class III GRPs with particular emphasis on *Arabidopsis* GRP17, are important. Class IV GRPs have a strong RNA binding moiety. Henceforth they are involved in molecular processes such as alternative splicing via spliceosome activity, or the regulation of transcription, and in circadian rhythm. Proteins from class IV play a role in the regulation of stomatal movement, seed development, and stamen development [Supplementary Tables 1a,b; GENEVESTIGATOR and eFP Browser data (Winter et al., 2007)].

Basing on the effects on cell division and cell elongation in an *Arabidopsis* mutant line with a *grp3-1* knockout, Mangeon et al. (2016) concluded that *At*GRP3 is involved in root size determination. The authors support evidence that, curiously, this mutant line presented enhanced Al tolerance (probably through the specific involvement of *At*WAK1-mediated signaling). Other data suggest that different *GRP* genes can have opposing roles in the cell elongation process, e.g., *At*GRP3 expression represses this process, while *At*GRP5 enhances it (Mangeon et al., 2009). Overall, these findings indicate the pleiotropic roles of GRPs in plant growth, development and stress responses (Table 2, Supplementary Tables 1, 2).

**Table 2:** Participation of the identified plant glycine-rich proteins in stress response.

***GRP* gene**	**Gene source**	**Stress response**	**Localization**	**References**
*At*GRP1	*Arabidopsis thaliana*	Overexpression of *At*GRP1 protein improve stress response in high-salt conditions	Nucleus/cytoplasm	Wang et al., 2012
*At*GRP2	*Arabidopsis thaliana*	Under cold stress GRP2 protein positively affects seed germination, cold inducible higher level of expression, predicted role in stabilization and modulation of mRNA in cold acclimation, up-regulation, GRP2 knockout mutants showed enhancement of germination after mannitol treatment, up-regulation enhances tolerance to drought stress	Nucleus/cytoplasm	Fusaro et al., 2007; Kim J. Y et al., 2007; Yang et al., 2014; Ciuzan et al., 2015
*At*GRP4	*Arabidopsis thaliana*	Up-regulation of abundance during cold adaptation process, overexpressed in high-salt conditions connected with the retarded seed germination	Nucleus/cytoplasm	Kwak et al., 2005, 2011; Kim J. Y et al., 2007
*At*GRP7	*Arabidopsis thaliana*	Up-regulation during cold adaptation process, involvement in the plant response under osmotic stress, influence on two stress-inducible genes *RD29A* and *RAB18*, under drought stress *GRP7* knockout mutants showed suppression of germination, up-regulation enhances tolerance to drought stress, upregulated also in response to peroxide-induced oxidative stress, increased level of *GRP7* transcripts enhances tolerance to biotic stress such as *Pectobacterium carotovorum*, tobacco mosaic virus and *Pseudomonas syringae*	Nucleus/cytoplasm	Cao et al., 2006; Fu et al., 2007; Kim J. S et al., 2007; Schmidt et al., 2010; Kwak et al., 2011; Lee et al., 2012; Yang et al., 2014; Ciuzan et al., 2015
*At*GRP7	*Arabidopsis thaliana*	RNA chaperone activity during the cold adaptation process, increased in abundance at low temperature, decreased in high temperature	Nucleus/cytoplasm	Kim J. S et al., 2007; Wienkoop et al., 2008; Cramer et al., 2011
*At*GRP8	*Arabidopsis thaliana*	Up-regulation under oxidative stress induced by the peroxide	Nucleus/cytoplasm	Schmidt et al., 2010
*At*RZ-1b	*Arabidopsis thaliana*	Bacterial cells overexpressing *At*RZ-1b grew well at low temperature, RNA chaperone activity	Nucleus/cytoplasm	Kim et al., 2010b
*Cs*GRP7-a	*Camelina sativa*	Plants with overexpression of GRP7 protein are more tolerant to cold and freeze treatment, negative role under high salinity conditions	Nucleus/cytoplasm	Kwak et al., 2016
*Hv*GRP2, *Hv*GRP3	*Hordeum vulgare*	Higher expression at low temperature (cold induced), involvement in the response to fungal pathogens *Erysiphe graminis* and *Rhynchosporium secalis*	Nucleus/cytoplasm	Molina et al., 1997
*Lb*GRP1	*Limonium bicolor*	Elevated proline content in *Nicotiana tabacum* plants under overexpression and the enhanced tolerance to salt stress	Nucleus/cytoplasm	Wang et al., 2012
*Lp*GRP1	*Lolium perenne*	Involvement in cold and freezing tolerance	Nucleus/cytoplasm	Shinozuka et al., 2006
*Ms*GRP	*Medicago sativa*	Plants with overexpression of GRPs are more sensitive to high salinity	Nucleus/cytoplasm	Long et al., 2013
*Ng*RBP	*Nicotiana glutinosa*	Induction by biotic stress and possible positive role in plant-pathogen interaction	Nucleus/cytoplasm	Naqvi et al., 1998
*Nt*GRP1	*Nicotiana tabacum*	*NtGRP1* transcripts were up-regulated after cold treatment in 40 day-old plants (without changes in expression in younger plants), up-regulation of these transcripts was also observed after wounding stress	Nucleus/cytoplasm	Khan et al., 2013
*Nt*GRP-1a, *Nt*GRP3	*Nicotiana tabacum*	Up-regulation under cold stress, under high temperature treatment and after wounding, under drought stress plants showed accumulation of *NtGRP-1a* transcripts and were more tolerant to these conditions	Nucleus/cytoplasm	Chen et al., 2010; Khan et al., 2013
*Os*GRP1, *Os*GRP4, *Os*GRP6	*Oryza sativa*	Plants with overexpression of those proteins are visibly more tolerant to the low temperature-related stress	Nucleus/cytoplasm	Kim et al., 2010a
*Pp*GRP3	*Physcomitrella patens*	Under low temperature stress *Pp*GRP3 protein is probably associated with post-transcriptional processing of mitochondrial RNA	Mitochondria	Nomata et al., 2004

### Tissue- and developmentally-specific expression of *Arabidopsis GRP* genes

*GRP* genes are expressed in a tissue- and organ-specific manner in *Arabidopsis* development. Class I accumulates mostly in seeds, siliques, roots, leaves, and also in the vegetative rosette and in the rosette after transition to the generative phase. The expression profile of GRPs from class II is similar; however, this is higher in cotyledons, hypocotyls, and cauline leaves. Class III is highly expressed in seeds, siliques, shoots, vegetative rosette, and flowers. This is associated with role of those proteins in lipid storage, sexual reproduction and pollen hydration or its recognition. However, the members of class IV are most highly expressed in shoot apex, vegetative rosette, seeds, and flowers. On the contrary, other *GRP* genes are lowly expressed in the most tested tissues and up-regulated in inflorescences. The expression of *GRP* genes is modulated in the key developmental stages, e.g., pollen and embryo development; however, most of them are expressed at low levels in pollen and relatively high levels in the most of tissues [eFP Browser (Winter et al., 2007) and RNA-seq data from Araport11 portal (Krishnakumar et al., 2015); Supplementary Tables 1b, 2a,b].

### Hormonal regulation of the expression of *GRP* genes

The regulation of adaptive responses at the level of gene expression can involve a plethora of molecular mechanisms. Some of the crucial steps in these processes are mediated by RBPs (Cao et al., 2006). GRPs from class I, II, and IV (notably subclass IVc) are involved in mediating responses to hormones, including abscisic acid (ABA), salicylic acid, or ethylene. ABA governs many important aspects of plant development and physiology; *GRP* expression often increases upon ABA treatment (Cao et al., 2006; Fusaro et al., 2007; Mangeon et al., 2010; Fu et al., 2016). Aneeta et al. (2002) reported that ABA (at 10 μM concentration) induced *sbGR-RNP* gene expression in *Sorghum bicolor*. Another gene involved in ABA signaling is *AtGRDP1*. 35S:*At*GRDP1 overexpressing lines were ABA-resistant whereas the disruption of *AtGRDP1* gene resulted in ABA hypersensitivity. Under ABA treatment *Atgrdp1-null* mutant seedlings showed higher level of *ABI3* and *ABI5* transcripts in contrast to 35S:*At*GRDP1 overexpressing lines in which these transcripts were repressed (Rodríguez-Hernández et al., 2014).

The altered expression of *GRP* genes under ABA treatment is connected with phenotypic modifications aiding stress responses. Cao et al. (2006) reported the repression of *AtGRP7* by ABA. In T-DNA mutants they observed hypersensitive responses to ABA evidenced both by the root growth and seed germination pattern. Notably, the authors found that *atgrp7-1* mutant plants accumulated *RD29A* and *RAB18* transcripts (both ABA- and stress-inducible) at higher abundance in comparison to wild-type plants. Therefore, *At*GRP7 is suspected of being involved in the regulation of ABA signaling in addition to stress responses. *AtGRP7* and *AtGRP8* respond to oxidative stress, and due to the fact that ABA results in both genes repression, negative feedback may be expected to occur between reactive oxygen species (ROS) and ABA signaling in this case (see section GRPs and Oxidative Stress Response; Carpenter et al., 1994; Cao et al., 2006). In addition, an *Arabidopsis* zinc finger containing GRPs (atRZ-1a) is involved in both stress responses and ABA signaling. Kim Y. O et al. (2007) used transgenic *Arabidopsis* plants, either overexpressing atRZ-1a or loss-of-function mutants, and observed that the overexpression of this gene resulted in retarded seedling germination and growth under salinity and drought in comparison to wild-type plants. In contrast, plants with loss-of-function mutations germinated earlier and grew faster than wild-type plants under the same stress conditions. However, the germination pattern of investigated transgenic and mutant plants was influenced by ABA or glucose; the results therefore indicate, that atRZ-1a affects germination in an ABA-dependent manner (Kim Y. O et al., 2007). Contrary to this, GRP2 affects seed germination through a ABA-independent pathway (Kim J. Y et al., 2007). No differences in germination between wild-type and transgenic plants following ABA or glucose addition (without cold exposure in parallel with ABA treatment) were observed. Such relationships between *GRP* transcript levels, hormone signaling, as well as plant growth and development were also supported by the findings of Ortega-Amaro et al. (2015). The results they obtained suggested that the deregulation of *AtGRDP2* expression in *Arabidopsis Atgrdp2* mutants and 35S:*At*GRDP2 overexpression lines results in plant developmental aberrations. *AtGRDP2* is a gene encoding a small GRP containing a DUF1399 domain and a putative RRM motif. *AtGRDP2* mRNAs mainly accumulate in flowers. Transgenic lettuce (*Lactuca sativa*) plants overexpressing *AtGRDP2* gene also manifest increased growth rate and early flowering time. The authors compared flowering time under long- and short-day conditions and observed faster development and early flowering in *At*GRDP2 overexpression lines in comparison with wild-type plants. Notably, under the same cultivation conditions, lines with *GRDP2* knockdown exhibited a late-flowering phenotype. This is clear evidence for the involvement of this gene during key developmental stages (Ortega-Amaro et al., 2015).

*At*GRDP2 expression influenced the expression of other genes, namely genes coding for auxin (IAA) response factors (*ARF2, ARF6, ARF8*) and *at-MIR167* involved in the control of floral development and auxin signaling. *ARF* gene expression is regulated by auxins and various IAA concentrations might be responsible for the more rapid growth of lines overexpressing *At*GRDP2. Two of the genes mentioned earlier (*ARF6* and *ARF8*) as well as *AUX1*, which encodes an auxin transporter, were induced in *35S::AtGRDP2-OE3* line; however, they were repressed in the *Atgrdp2-1* mutant. In contrast, *ARF2* expression was induced in *Atgrdp2-1* line. The level of *miR167*, which is a negative regulator of *ARF6* and *ARF8*, was increased in *Atgrdp2-1* mutant and decreased in *AtGRPD2* overexpressing line (Ortega-Amaro et al., 2015). These results reveal the important roles of *AtGRDP2* in development and the stress response; interestingly, they suggested a connection between *AtGRDP2* gene expression pattern and hormone signaling. However, the involvement of *GRP* gene expression in the action of hormones and signaling pathways needs to be further investigated.

### Expression of *GRP* genes across stress treatments-general remarks

GRPs from classes I, II, and IV (especially subclass IVc) are involved in various stress responses (Kar et al., 2012); subclass IVc participates notably in cold acclimation. The important role of the distinct RZ class of GRPs in such responses has been also established in *Arabidopsis*, rice and wheat (*Triticum aestivum*) (Kim et al., 2005, 2010a,b; Xu et al., 2014). According to the GENEVESTIGATOR and *Arabidopsis* eFP Browser data (Supplementary Tables 1a, 2c,d; Winter et al., 2007), selected *Arabidopsis GRP* genes (e.g., *MCO15_15*) respond to ozone, UV irradiation and Fe deficiency (e.g., *MCO15_15* and *AtGRP3S*) by increasing of the abundance of their respective mRNAs. Under stress, up-regulation of *GRP* genes exceeds down-regulations. In Supplementary Tables 1, 2 the various expression patterns of GRPs from different classes are compiled according to hormonal regulation, developmental stage as well as to abiotic and biotic stimuli.

Despite the findings discussed above, data on the expression patterns of *GRP* genes under unfavorable conditions are nor comprehensive. This is discussed further in subsequent sections The Role of GRPs in Temperature Stress, GRPs in Mechanical Stress, Participation of GRPs in Salinity/Osmotic Stress Responses, Participation of GRPs in Drought Stress, GRPs and Oxidative Stress Response, and GRPs in Biotic Stress Responses.

## The role of GRPs in temperature stress

According to the literature, three distinct subtypes of temperature-related stress acting on plant cells at the physiological and molecular level can be distinguished. The first occurs at temperatures below freezing, the second acts at low (but above freezing) temperatures (0–20°C), and the third refers to high-temperature treatments (Iba, 2002; Sinha et al., 2015). To date, some GRPs have been described mainly as proteins that enhance plant tolerance to low temperatures. Moreover, it has been suggested that they are involved in cold acclimation and may improve plant growth at low temperatures. In recent years, however, a plethora of novel data on the participation of GRPs in responses to altered temperature has been reported and is presented below.

### GRPs and low temperature treatments

Low temperature treatments influence growth, development, productivity, and geographical distribution of crops (Sanghera et al., 2011; Zhou et al., 2011; Yang et al., 2015; Fu et al., 2016). In general, most plant species adapted to tropical and subtropical zones are highly intolerant to chilling and freezing; however, plants grown in temperate climates can adapt to lower temperatures (“cold acclimation”; Xin et al., 2013; Zhan et al., 2016).

GRPs were reported to be involved in acclimation to various temperatures (Table 2). Notably, in *Arabidopsis* leaves and roots, the expression of *GRP* genes responded sensitively to cold treatment (Supplementary Table 2; Kim J. S et al., 2007; Khan et al., 2013; Mangeon et al., 2016). According to Kim Y. O et al. (2007), several members of the GRP superfamily confer a significant impact on *Arabidopsis* seed germination and seedling growth under stress, including not only suboptimal temperature treatments, but also salinity or water deficiency. However, the upregulation of *GRP1* gene, which was well visible after cold stress in tobacco plants [*Nicotiana tabacum*; 4°C treatment at 16:8-h light:dark (L:D) photoperiod, 20, 30, and 40 days after germination] cannot indicate the true relationship between *GRP1* and decreased temperature treatment, as it was applied in laboratory conditions to a subtropical plant species and thus without any particular environmental relevance (Khan et al., 2013). In response to cold, the authors showed variations in *NtGRP1* mRNA abundance across tobacco plant development (the highest was in 40-day-old plants).

The level of *GRP1* transcripts in crowns, roots and leaves of perennial ryegrass (*Lolium perenne*) was significantly increased in the course of cold acclimation (6/2°C 8:16-h L:D photoperiod). Over the next 1 week of cold acclimation, the abundance of *GRP1* messengers rose further. However, the increased level of *LpGRP1* transcripts remained unchanged under prolonged cold stress. When plants were transferred to the control temperature, de-acclimation of freezing tolerance accompanied by the reversion to control level of *GRP1* mRNAs was observed (Table 2; Shinozuka et al., 2006). Notably, perennial ryegrass belongs to important pasture grasses having ability to acquire cold tolerance during exposure to low, but non-lethal temperatures.

Kim J. S et al. (2007) reported that *GRP2* gene was induced and that *GRP4* and *GRP7* were also upregulated by *Arabidopsis* chilling (4°C for 1–4 days under a 16-h photoperiod; Table 2); they also found enhanced freezing tolerance of *Arabidopsis* plants with GRP2 overexpression compared with the wild type and *grp2*-knockout mutants. Under cold stress, GRP2 enhances seed germination at low temperature (11°C) and influences *Arabidopsis* growth in ABA-independent manner (Kim J. Y et al., 2007). Fusaro et al. (2007) observed a strong induction of *AtGRP2* gene expression after 6 h of cold treatment (6°C, 16/8-h L/D photoperiod). A higher level of expression was detected till the end of 48-h-long treatment. In this study an early cold-response was noted as the upregulation of *AtGRP2* gene was associated with its cold- inducible activity. Fusaro et al. (2007) suggested that plant GRPs are involved in a specific mechanism active in the cold response that acts by stabilizing various transcripts under cold treatment (GRP2 has a strong *in vitro* affinity to ssRNA and DNA, including homopolymers).

Furthermore, Kim et al. (2010) demonstrated the complementation of a cold-sensitive *E. coli* mutant by *At*RZ-1b during cold treatment (18°C) of bacterial cells. *E. coli* overexpressing *At*RZ-1b grew well at the lowered temperature; colony forming ability was comparable to that of cells overexpressing bacterial RNA chaperone CspA (Table 2), which suggests that *At*RZ-1b possesses RNA chaperone activity. In contrast, *At*RZ-1c appeared unable to complement cold sensitivity in the same bacterial strain (Kim et al., 2010b).

The ability to suppress cold stress appeared not to be equal for all the investigated *GRP* genes. For instance, the *GRP7* gene appeared to have a greater protective effect during the cold response than *GRP2*. The GRP7 protein also has the strongest ability to increase *E. coli* viability or growth rate under cold shock at 4 or 17°C, respectively, and its RNA chaperone activity during the cold adaptation process was shown (Kim J. S et al., 2007). The results obtained by the authors suggest that plant and bacterial cold shock proteins (CSPs) play similar roles in the positive regulation of cell growth at low temperature. Interestingly, the activity of GRPs under low-temperature conditions may depend on the number and size of the C-terminal glycine-rich region (GRP4 has a shorter region in comparison with GRP7 or GRP2 and displays the lowest chaperone activity). Kwak et al. (2011) suggested a crucial role for the N-terminal sequence of *At*GRP4 and *At*GRP7 in RNA chaperoning; the overall secondary structure of GRPs (including the presence of all RRMs) may be critical for cold adaptation. However, more data is needed to confirm this relationship.

Further interesting results were obtained from the complementation of CSP knockouts in the *E. coli* BX04 cold-sensitive mutant with *GRP* genes from *Arabidopsis* and rice. The BX04 strain has 4 genes coding for CSPs deleted; thus, it is extremely intolerant to low-temperature treatments. Notably, Kim et al. (2010a) concluded that specific types of *Os*GRPs are capable of increasing of cold-tolerance of this strain (Table 2). The authors clearly demonstrated RNA chaperone activity during cold adaptation for the investigated GRPs.

Kim et al. (2010a) showed that rice GRPs (*Os*GRP1, *Os*GRP4, and *Os*GRP6) have ability to rescue *grp7*-*Arabidopsis* knockout plants from cold, and *Arabidopsis* plants overexpressing these proteins were more tolerant to low temperatures (*Arabidopsis* seedlings were cold-stressed at 8-11°C). It is worth mentioning that the authors determined the expression profiles of 6 diverse *OsGRP* genes, which correlated well with the ability to complement cold sensitivity in *E. coli* mutant. *Os*GRP2 appeared to exhibit the lowest accumulation and did not provide cold sensitivity complementation. In contrast, *OsGRP1* and *OsGRP6* showed the highest complementation ability and at the same time displayed higher mRNA accumulation than *OsGRP2*.

Another example of the involvement of GRPs in stress responses during cold treatment might be *Nt*GRP-1a and *Nt*GRP-3 transcripts (Table 2). These transcripts appeared to increase in abundance 1h after the induction of cold stress (4°C treatment of 4-week-old plants), then slightly decreased after 2 h, then showed continuous upregulation after 4 h (Chen et al., 2010). The same conditions applied to 13-day-old barley (*Hordeum vulgare*) seedlings grown at 22/18°C L:D thermal regime and 16:8 h L:D photoperiod also led to a significant increase in the abundance of selected *GRP* mRNAs. In this case, a higher level of expression of cold-inducible *Hvgrp2* and *Hvgrp3* genes was also noted (Molina et al., 1997).

Kwak et al. (2016) presented further evidence supporting the important role of GRPs in low temperature stress responses; transgenic *Camelina sativa* plants with *CsGRP7a* overexpression appeared to be cold-tolerant. *C. sativa* is a fast growing crop with low requirements for fertilization. After the transgenic 14-day-old *C. sativa* plants were exposed to cold stress (2°C, 16:8-h L:D photoperiod; Kwak et al., 2016), they had improved survival parameters compared to wild-type plants or *grp7* knockout plants (Table 2), supporting the important role of *GRP7* during cold responses and cold acclimation.

Notably, GRP7 accumulation is regulated by the circadian clock via the autosplicing of *Atgrp*7 pre-mRNA (a feedback loop). Staiger et al. (2003) suggested a role of *At*GRP7 in splice site selection. In transgenic *Arabidopsis* plants, the constitutive *At*GRP7 overexpression is strictly connected with the accumulation of low amounts of alternatively spliced *Atgrp7* mRNAs containing a premature stop codon (due to the usage of 5′cryptic splice site in the intron by *At*GRP7). The alternatively spliced transcripts do not accumulate at high levels due to their instability; henceforth, they do not produce functional GRP7 protein. When *At*GRP7 is overexpressed, it interacts with 3′UTR and the intron of *Atgrp7* messenger. *At*GRP7 influences also the choice of the splice site within the *Atgrp8* transcript encoding a glycine-rich RNA-binding protein related to *At*GRP7. This is attributed to the favorable accumulation of the unstable *Atgrp8* transcript, which is alternatively spliced. Such results unambiguously indicate that the mentioned regulatory mechanism is crucial for controlling of GRP levels and that *At*GRP7 can efficiently regulate the abundance of target messengers (Staiger et al., 2003).

Further insights into functions and interacting partners of some GRPs were provided by a pioneering study by Meyer et al. (2017) involving the application of an individual nucleotide resolution crosslinking and immunoprecipitation (iCLIP) approach for the identification *in vivo* targets of *At*GRP7. *Arabidopsis* plants overexpressing GRP7 fused with GFP were used for iCLIP experiments that allowed the identification of 858 messengers with enriched crosslink sites. They were absent in plants containing variants of GRP7 with dysfunctional RRMs or in control lines expressing GFP only. Moreover, an RNA immunoprecipitation (RIP)-sequencing strategy led to the validation of 452 highly-confident binders (constituting for 53% of iCLIP targets). The iCLIP and RIP pools of targets were thus non-identical, but partially overlapped. The abundance and splicing some of some of those transcripts were regulated by *At*GRP7. Overall, the results of Meyer et al. (2017) highlight the importance of the RNA binding motif in controlling multiple *At*GRP7 targets. It appeared that *At*GRP7, which is controlled by the circadian clock, preferentially binds to 3′UTRs. *At*GRP7 overexpression notably represses the expression of target genes and affects circadian oscillations (e.g., *CCL* and *DRM2* targets), alternative splicing, as well as the polyadenylation of selected messengers. In addition, *At*GRP7 could also bind diverse regions of the same transcripts, which suggests its diverged function. Among the high-confidence binders of *At*GRP7, cold-, salinity-, and pathogen defense-responsive transcripts were distinctive. However, the role of GRP7 in tolerance to such stress conditions may depend on various regulatory mechanisms without a direct relationship with mRNA abundance. These results pinpoint the necessity of studying the reconfiguration of post-transcriptional networks (in which GRP7 is involved) under adverse conditions.

Finally, mitochondrial GRPs seem to be also involved in responses to cold stress. Owing to their function, mitochondrial GRPs represents a unique subgroup of plant GRPs. Under low temperature, *Physcomitrella patens GRP3* transcripts and mitochondria-targeted GRP3 protein accumulated markedly; it was speculated that this protein may be associated with RNA splicing and editing during stress. The experiment was performed on 7-day-old protonemata of *P. patens* which had grown at 22°C under continuous light then transferred to 4°C for 12, 24, 36, and 48 h (Nomata et al., 2004). Due to the fact that some GRPs are present in *P. patens*, it might be obvious that mitochondrial GRPs (many of which remain unidentified) must have separated from another plant GRP superfamily at an early stage of plant evolution (Nomata et al., 2004).

### GRPs and heat stress

Elevated temperatures exert a negative impact on plant growth, development and productivity (Zhu et al., 2013; Hu et al., 2015; Zhai et al., 2016). Heat stress during the generative phase of food crop growth might be particularly destructive. Recent results have supported the important role of these proteins in the acquisition of heat tolerance. Zhu et al. (2013) identified four down-regulated protein spots containing GRPs in heat-stressed *Pinellia ternata* (38°C, 1 day), which indicated possible metabolic impairment under stress. The level of *GRP* transcripts in *P. ternata* leaves after various durations of stress was not reflected by the protein accumulation profile. At the beginning of heat stress, *GRP* transcripts were slightly down-regulated, but after 12 h they rose significantly, which may be associated with post-transcriptional and post-translational events. In tobacco Chen et al. (2010) observed a remarkable increase in the abundance of *NtGRP-1a* and *NtGRP-3* transcripts, with a peak at after 8 h of heat stress. Under the same conditions, curiously, the expression of *NtGRP-1b* gene was unaffected.

Wienkoop et al. (2008) also demonstrated the involvement of *At*GRP7 in temperature stress responses (Table 2). The level of *At*GRP7 protein was high at low temperatures, compared with elevated temperature (32°C, 3 days) (Supplementary Table 2). *At*GRP7 abundance correlated with proline and glutamine content. Interestingly, the temperature response of *At*GRP7 as well as osmolyte accumulation appeared independently of the responses of other markers characteristic of temperature stress.

## GRPs in mechanical stress

GRPs perform essential roles in plant survival and respond to mechanical stress, including wounding after pathogen infection. Such responses may be associated with increased membrane permeabilization and, consequently, to pathogen cell death (Cândido Ede et al., 2011; Boyd et al., 2013; Savatin et al., 2014). Khan et al. (2013) measured *NtGRP1* mRNA expression levels after leaf wounding by scraping with pins and observed a 2.5-fold increase 12 h after treatment. However, *NtGRP1* expression levels decreased to control values 24 h after the cessation of stress. Similar results in tobacco were reported by Chen et al. (2010). *NtGRP-1a* and *NtGRP-3* genes were rapidly upregulated at 1 h after wounding (cutting leaves with forceps), then their expression increased until reaching a peak at 4 h-long stress and gradually reducing (Table 2).

In summary, the exact role of GRPs during heat and mechanical stress has to be elucidated and the mode of its action remains unclear.

## Participation of GRPs in salinity/osmotic stress responses

Plants respond to salinity stress at various levels. This type of abiotic stress can be particularly detrimental, because of protein denaturation, ionic imbalance, and toxicity, ROS production and subsequent osmotic stress, loss of turgor, as well as membrane modifications and other cellular effects (Conde et al., 2011; Long et al., 2013; Joshi et al., 2014; AbdElgawad et al., 2016; Kushwaha et al., 2016). Salinity stress can cause abnormalities during enzymatic reactions, due to Na^+^ toxicity and elevated K^+^ uptake (Suzuki et al., 2016). Elevated levels of salt in the soil may initiate drought stress; moreover, the coexistence of salinity and drought is very frequent. GRPs have a suggested influence on *Arabidopsis* seed germination under salinity (75, 100, 125, 150, and 175 mM NaCl), but strikingly without any apparent influence on seedling growth. GRP2 seems to play a significant role in seed germination and seedling growth of *Arabidopsis* plants under osmotic stress (Kim J. Y et al., 2007).

Under high-salt conditions (250 mM NaCl), the overexpression of GRP4, another GRP family member identified in *Arabidopsis*, retarded seed germination in this species (Table 2). The same was observed by Kwak et al. (2005) under dehydration (4-week-old plants were placed on a filter paper for 1 h at room temperature to remove residual water completely, and then were transferred to a growth chamber at 23°C).

Another GRP involved in the osmotic stress response is GRP7 (Tables 2, **3**). The expression of the *AtGRP7* gene was repressed by 300 mM NaCl; the application of very high concentrations of NaCl in Kwak et al. (2005) and Cao et al. (2006) studies may result in sub-lethal effects associated with the massive repression of gene expression, however salinity stress in the Cao et al. (2006) report lasted for 6 h only. On the contrary, increased levels of expression of *AtGRP7* observed during seed germination suggested hypersensitive responses to osmotic stress conditions. Moreover, the *atgrp7-1* mutant plants lacking *AtGRP7* gene, presented significantly higher *RD29A* and *RAB18* transcripts level in comparison to wild-type plants. This evidence supports the involvement of *AtGRP7* in plant osmotic stress response (Cao et al., 2006). Kwak et al. (2016) also proposed a negative role of *Cs*GRP7 under high salinity. During salt stress, the growth of transgenic *C. sativa* plants was retarded in comparison with wild-type plants. Using specific *Medicago sativa* expressed-sequence tag data, Long et al. (2013) cloned and characterized a novel *MsGRP* gene (accession no. JQ340083) and suggest its negative role in salinity responses as *Arabidopsis* plants overexpressing *M. sativa* protein appeared to be more sensitive to high salinity than wild-type plants.

Ortega-Amaro et al. (2015) compared salt stress tolerance in *Arabidopsis* wild-type plants, *Atgrdp*2 mutants and 35S:*At*GRDP2 overexpression lines. *Arabidopsis* lines with GRDP2 overexpression displayed enhanced tolerance to high salinity conditions. After 7 days of 150 mM NaCl treatment they observed that more than 50% plants with *At*GRDP2 overexpression recovered in comparison with only 20% in the case of *Atgrdp2-1* mutant.

Additional clues about the involvement of GRPs in salinity and osmotic stress tolerance were obtained by Rodríguez-Hernández et al. (2014). *At*GRDP1, like *A*tGRDP2, contains DUF1399 domain, a putative ribonucleoprotein (RNP) motif and a short glycine-rich domain. The authors showed that the expression of *At*GRDP2 is modulated in response to mannitol, sorbitol, glucose, NaCl, LiCl, and ABA treatment. The *AtGRDP1* gene expression level was greatly increased depending on the quality and duration of treatment in comparison to control plants. On the contrary, *Atgrdp1-null* mutant line displays increased sensitivity to salt and osmotic stress during cotyledon development and germination.

During stress, plants have the ability to accumulate diverse osmolytes controlling osmotic potential inside cells. There is evidence for a positive correlation between proline accumulation and the participation of GRPs in salinity responses. Wang et al. (2012) demonstrated that the overexpression of *Lb*GRP1 (from *Limonium bicolor*) in transgenic tobacco plants can markedly increase proline content under salt stress (Table 2). They showed that the transgenic lines displayed higher salt tolerance than wild-type plants indicating a significant improvement in stress responses under high salinity that was attributable to *At*GRP1 overexpression. Aneeta et al. (2002) measured the level of *Sb*GR-RNP (GRP from *Sorghum bicolor* containing the conserved ribonucleoprotein motif) after 1 day treatments with 1 M and 500 mM NaCl. They observed a notable increase in *SbGR-RNP* transcripts abundance in *S. bicolor* seedlings subjected to all tested stress conditions.

Taken together, these findings markedly enhanced current knowledge of the functional roles of GRPs in response to salinity and osmotic stress.

## Participation of GRPs in drought stress

Drought often results in stomatal closure reduction, decrease in stomatal density and/or increased water uptake (Yu et al., 2008, 2013; Zhu et al., 2016). Dehydration and subsequent rehydration might induce “transcriptional memory,” helping plants to mitigate harmful effects (Chinnusamy and Zhu, 2009; Ding et al., 2012; Hu et al., 2015).

Ciuzan et al. (2015) tested the influence of drought stress on *Arabidopsis* seed germination and showed that *GRP2* knockouts (contrary to *GRP7*) showed enhanced germination after mannitol treatment (Table 2). Yang et al. (2014) also examined the influence *At*GRP2 and *At*GRP7 overexpression on drought tolerance in transgenic rice. Contrary to the findings of Ciuzan et al. (2015), Yang et al. (2014) indicated that rice plants overexpressing the mentioned GRPs were more tolerant to water deficit than wild-type plants, which may illustrate species-specific effects of GRP overexpression. Chen et al. (2010) measured the accumulation of *NtGRP-1a* transcripts under drought stress (seedlings without watering in a growth chamber with 50% humidity and 16:8-h L:D photoperiod) and demonstrated that they were continuously accumulated for 3–6 days (Table 2). Notably, plants died if drought time lengthened beyond 6 days.

## GRPs and oxidative stress response

Biotic and abiotic stress factors can lead to the rapid production of ROS in plant tissues, which can cause extensive damage to cell membranes and other cellular components. The disproportionate production of ROS relative to scavenging rate results in oxidative stress and represents one of the most common causes of stress-induced injuries (Tuteja et al., 2014). Plants have evolved a number of effective mechanisms for ROS detoxification (Lehmann et al., 2012).

The impact of oxidative stress on the expression patterns of *GPR* genes has not been extensively investigated. Schmidt et al. (2010) noticed alterations in the expression profiles of *AtGRP7* and *AtGRP8* genes during oxidative stress (Table 2). Both genes were rapidly upregulated in response to peroxide-induced oxidative stress in *Arabidopsis* plants, which is in line with results obtained for these genes in response to other stresses (see above). The negative relationship between oxidative stress and ABA signaling may be particularly expected in this case as Cao et al. (2006) and Carpenter et al. (1994) demonstrated that *AtGRP7* and *AtGRP8* expression is downregulated (even 2- to 3-fold) by ABA treatment.

## GRPs in biotic stress responses

Biotic stress, including bacterial, fungal, nematode, and virus infections might be also fatal for plants. Climate change influences pathogen spread and infection intensity. Biotic and abiotic stress conditions (drought, in particular) acting together result in particularly destructive effects on plant growth and development; they also accelerate further pathogen invasion (Suzuki et al., 2014; Sinha et al., 2017).

In *Nicotiana glutinosa*, the expression of a gene encoding glycine-rich RNA-binding protein (*Ng*RBP) containing two conserved N-terminal RNP motifs as well as C-terminal glycine-rich domain, was negatively regulated under tobacco mosaic virus (TMV) infection; at 24 h after TMV infection, an increased level of *ngRBP* mRNAs was observed (Table 2). This observation suggests that *Ng*RBP protein is induced by a specific biotic stressor and may play an important role in plant-pathogen interactions (Naqvi et al., 1998). The expression of *Hvgrp2* and *Hvgrp3* genes in barley was also altered during infection by fungal pathogens *Erysiphe graminis* and *Rhynchosporium secalis* (Molina et al., 1997).

Lee et al. (2012) provided further evidence for the participation of *At*GRP7 in pathogen response and defense (Table 2). Due to its role in the regulation of RNA metabolism, *At*GRP7 is generally important for plant innate immunity; in addition, this protein seems to be a specific “defense regulator.” *AtGRP7* transcript levels were significantly elevated in *Arabidopsis* plants 48 h after *Pectobacterium carotovorum* infection. Interestingly, *AtGRP7* transcript levels upon *B. cinerea* infection were not substantially altered. This might suggest a species-specific mode of action and different effects of this protein, depending on the pathogen type (Lee et al., 2012).

In summary, it has been shown that *At*GRP7 plays a positive role in defense against *P. carotovorum* in *Arabidopsis* and in transgenic tobacco plants against TMV. Wild-type *Arabidopsis* plants exhibited greater resistance to the growth of *P. carotovorum* than in the case of *grp7* mutant plants. For transgenic tobacco lines with overexpressed *At*GRP7 protein, necrotic lesion formation was delayed and the total number and size of these lesions was significantly less than in wild-type plants (Lee et al., 2012). However, contrasting results were obtained by this research group regarding the role of *At*GRP7 in defense against *B. cinerea*, because this protein enhances the susceptibility of *Arabidopsis* plants to this fungus. This result is correlated with stable *AtGRP7* transcript levels and confirms that GRP7 protein is a negative “defense regulator” (Lee et al., 2012).

Other interesting results confirming a positive role for *At*GRP7 in plant pathogen defense were obtained by Fu et al. (2007). They showed that *Arabidopsis grp7* mutants (with *At*GRP7 suppressed expression) were much more susceptible to *P. syringae* than wild-type plants (Fu et al., 2007).

## Future directions and concluding remarks

Plants are organisms without any active ability to change their environment under unfavorable conditions. Consequently, plants have evolved various adaptive mechanisms that enable them to cope with stress. In the natural environment, plants rarely encounter single stressors; in fact, plant stress responses depend on stressor combinations. Glycine-rich RBPs are among the most crucial proteins responding to such conditions. To date, data available in the literature supported their significant role in response to various stress conditions.

There is a growing body of evidence indicating that protein engineering and biotechnology is an important tool for the production of plant cultivars with improved stress resistance. In the future, the isolation, cloning, characterization and functional validation of diverse GRPs that are expressed in response to various stresses might be helpful in achieving this goal. As discussed in the present review, the experimental results describing involvement of GRPs in plant stress responses suggest that the overexpression of these proteins may play a positive role in plant adaptation response to a range of stressors. Plant species displaying increased levels of *GRP* transcripts were more stress-resistant than wild-type plants. According to functional analyses, a role for GRPs in the stress response in many plant species, including model and non-model ones, is indicated. However, relatively little is known about the mechanisms underlying these processes, and this gap in knowledge should be promptly bridged. For instance, the question posed by Mangeon et al. (2010) on the participation of GRPs in multifunctional protein complexes as potent interactors is still open. To what extent could GRPs be replaced by other proteins assisting in signaling pathways within the plant cell? How do the functions of GRPs differ between monocotyledons and dicotyledons? In addition, the relevance of additional structural domains found among GRPs is far from understood. Can they replace glycine-rich domains functionally? It would be also valuable to better understand how glycine-rich domains affect the function of GRPs. Current research regarding GRPs (especially in the functional context) is still growing, and this superfamily indeed seems to have a prospective role in agriculture and consequently in worldwide food production.

## Author contributions

MC: Designed the review, deposited and analyzed all literature data, prepared Figure 1, Tables 1, 2 and wrote the manuscript; MR: Substantially assisted in the designation of the review concept, prepared supplementary materials, reviewed the data quality and soundness as well as updated the entire manuscript. Both authors have accepted the final version of the manuscript and agreed to be accountable for all aspects of the work.

### Conflict of interest statement

The authors declare that the research was conducted in the absence of any commercial or financial relationships that could be construed as a potential conflict of interest. The reviewer IW and handling Editor declared their shared affiliation.
